# Phenylbutyrate modulates polyamine acetylase and ameliorates Snyder-Robinson syndrome in a *Drosophila* model and patient cells

**DOI:** 10.1172/jci.insight.158457

**Published:** 2022-07-08

**Authors:** Xianzun Tao, Yi Zhu, Zoraida Diaz-Perez, Seok-Ho Yu, Jackson R. Foley, Tracy Murray Stewart, Robert A. Casero, Richard Steet, R. Grace Zhai

**Affiliations:** 1 Department of Molecular and Cellular Pharmacology, University of Miami Miller School of Medicine, Miami, Florida, USA.; 2 JC Self Research Institute, Greenwood Genetic Center, Greenwood, South Carolina, USA.; 3 Sidney Kimmel Comprehensive Cancer Center, Johns Hopkins School of Medicine, Baltimore, Maryland, USA.

**Keywords:** Genetics, Therapeutics, Genetic diseases, Lysosomes, Polyamines

## Abstract

Polyamine dysregulation plays key roles in a broad range of human diseases from cancer to neurodegeneration. Snyder-Robinson syndrome (SRS) is the first known genetic disorder of the polyamine pathway, caused by X-linked recessive loss-of-function mutations in spermine synthase. In the *Drosophila* SRS model, altered spermidine/spermine balance has been associated with increased generation of ROS and aldehydes, consistent with elevated spermidine catabolism. These toxic byproducts cause mitochondrial and lysosomal dysfunction, which are also observed in cells from SRS patients. No efficient therapy is available. We explored the biochemical mechanism and discovered acetyl-CoA reduction and altered protein acetylation as potentially novel pathomechanisms of SRS. We repurposed the FDA-approved drug phenylbutyrate (PBA) to treat SRS using an in vivo *Drosophila* model and patient fibroblast cell models. PBA treatment significantly restored the function of mitochondria and autolysosomes and extended life span in vivo in the *Drosophila* SRS model. Treating fibroblasts of patients with SRS with PBA ameliorated autolysosome dysfunction. We further explored the mechanism of drug action and found that PBA downregulates the first and rate-limiting spermidine catabolic enzyme spermidine/spermine N^1^-acetyltransferase 1 (SAT1), reduces the production of toxic metabolites, and inhibits the reduction of the substrate acetyl-CoA. Taken together, we revealed PBA as a potential modulator of SAT1 and acetyl-CoA levels and propose PBA as a therapy for SRS and potentially other polyamine dysregulation–related diseases.

## Introduction

Polyamines, including spermidine, spermine, and their precursor putrescine, are tightly regulated polycationic molecules that are broadly involved in cellular activities (Supplemental Figure 1; supplemental material available online with this article; https://doi.org/10.1172/jci.insight.158457DS1) (1–3). Polyamine metabolic dysregulation has been intensively investigated in pathological conditions, including aging, cancers, and neurological and immunological diseases (4–17). Gene mutations of polyamine pathway enzymes cause severe diseases (18–22). As the first confirmed genetic disorder associated with the polyamine metabolic pathway, Snyder-Robinson syndrome (SRS) is caused by X-linked recessive loss-of-function mutations in *spermine synthase* (*SMS*) (21, 22). SRS is a complex, multisystem disease characterized by a collection of clinical features including mild to severe intellectual disability, hypotonia, skeletal defects, movement disorders, speech and vision impairment, seizures, and cerebellar circuitry dysfunction (21, 23, 24).

SMS catalyzes the conversion of spermidine to spermine. In cells with loss of SMS function, the conversion of spermidine to spermine is inefficient, resulting in an accumulation of spermidine and an increase of the spermidine/spermine ratio. The absolute level of spermine is not consistently reduced, however, likely due to the compensation from intake from the extracellular environment (22). In the *Drosophila* model of SRS, accumulated spermidine leads to increased catabolism and back conversion to its precursor putrescine, accompanied by a production of the catabolites ROS and aldehydes (22). High levels of ROS and aldehydes have been shown to damage cellular organelles, such as mitochondria and lysosomes, which are impaired in the cells derived from patients with SRS, resulting in neuronal toxicity (22). Although the aldehyde from acetylated polyamine catabolism, 3-acetamidopropanal, was reported to be nontoxic in cultured cells, whether it spontaneously degrades to more toxic 3-aminopropanal or acrolein in tissues remains to be determined (25, 26).

Currently, treatment for SRS is limited. Some manifestation treatments show slight improvement in some patients, such as calcium supplementation to treat osteoporosis (27). Antioxidant treatment, such as the ROS scavenger AD4, partially restores mitochondrial function but not lysosomal function in the *Drosophila* model (22). Certain polyamine analogs also show some benefit in rebalancing the spermidine/spermine ratio in cell lines and disease models (28, 29). Efforts to rebalance the spermidine/spermine ratio by direct spermine supplementation in vivo have not been beneficial (28–30).

As the first and rate-limiting enzyme of spermidine catabolism, spermidine/spermine N^1^-acetyltransferase 1 (SAT1) acetylates spermidine with acetyl-CoA as the donor of the acetyl group (31). SAT1 has been shown to play key roles in hair growth, osteoblastogenesis, lipid metabolism, glucose metabolism, antiviral immunity, cancer, and neurodegeneration (12, 32–42). Overexpression of SAT1 causes transient depletion of spermidine and chronic reduction of acetyl-CoA, possibly resulting from futile polyamine cycling, in which spermidine is constantly synthesized from its precursor putrescine, immediately acetylated by SAT1 and then back-converted to putrescine (34, 36). SAT1 expression level is tightly regulated from transcription and translation to stability (31, 43–47). Given its critical role in polyamine catabolism, SAT1 could be a potential drug target for therapeutic considerations for SRS.

As a precursor molecule of phenylacetyl-CoA, phenylbutyrate (PBA) has been used as an alternative ammonium-removing agent for urea cycle disorder treatment (48). PBA-derived phenylacetyl-CoA interacts with glutamine to form phenylacetyl-glutamine, which is excreted through urine (49). PBA has also been broadly investigated as a chemical chaperone in protein misfolding or mislocalization-related diseases, as well as a histone deacetylase (HDAC) inhibitor in aging or cancer (50–55). Beneficial effects of PBA on the neurological system have been observed, although the detailed mechanisms remain to be explored (56–61).

In this study, we repurposed PBA for SRS treatment using an in vivo *Drosophila* model and cultured cells of patients with SRS. We revealed potentially new roles of PBA on polyamine catabolism and protein acetylation regulation. This identified activity of PBA remarkably reduced the toxicity of dysregulated polyamine metabolism and restored impaired cellular functions associated with the SRS condition.

## Results

### PBA attenuates SAT1.

**Figure 1:**
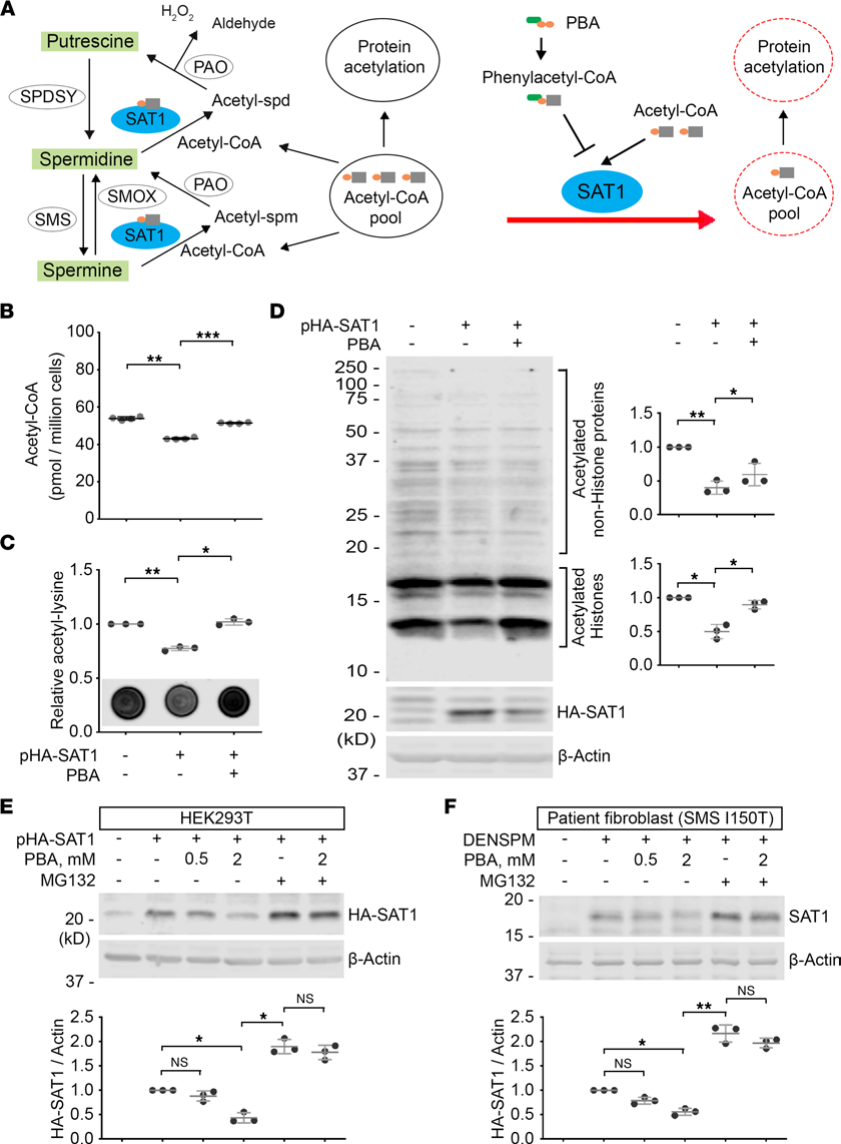
PBA attenuates SAT1. (**A**) Diagram of the hypothesis of PBA-derived phenylacetyl-CoA competing with acetyl-CoA to interfere with SAT1-mediated polyamine catabolism and acetyl-CoA consumption. SPDSY, spermidine synthase; PAO: peroxisomal N^1^-acetyl-spermine/spermidine oxidase; SMOX, spermine oxidase. (**B**) Acetyl-CoA level in HEK293T cells with SAT1 overexpression with or without 2 mM PBA treatment. Cells from 4 separate experiments were stored at –80°C and then tested in a single plate. *n* = 4; 1-way ANOVA multiple comparisons (matched). (**C**) Dot blot of acetyl-lysine in HEK293T cells with SAT1 overexpression with or without 2 mM PBA treatment. The values of the quantification were normalized with the control samples. (**D**) Western blot of the samples in **C**. Acetylated histones and nonhistone proteins in the bracket areas were quantified separately and normalized with β-Actin level. All values were further normalized by the control samples. (**E**) Western blot of HA-tagged SAT1 overexpressed in HEK293T cells with PBA, MG132, or the combination treatment. The HA-SAT1 level was normalized with the β-Actin level. The values of the cells without HA-SAT1 plasmid transfection were set as background. All the values were further normalized by that of the cells with indicated treatment. (**F**) Western blot of SAT1 induced by DENSPM in patient fibroblasts (CMS1849a) with indicated treatment. The SAT1 level was normalized with the β-Actin level. Values of the cells without DENSPM treatment were set as background. All values were further normalized by that of cells with DENSPM and without PBA or MG132 treatment. Images in **C**–**F** are representative of 3 separate experiments. *n* =3; *P < 0.05, **P < 0.01, ***P < 0.001; 1-way ANOVA multiple comparisons (matched). Data represent mean ± SEM.

Since acetyl-CoA binds to SAT1 as the donor of the acetyl group (43), we hypothesized that acetyl-CoA analogs, such as phenylacetyl-CoA, could competitively inhibit SAT1 (Figure 1A) in a manner similar to phenylacetyl-CoA competitively inhibiting choline acetyltransferase (62). The FDA-approved drug PBA is a promising prodrug candidate, because it has been shown to be catabolized to phenylacetyl-CoA very efficiently in vivo (48). To determine the effect of PBA on the cellular activity of SAT1, we overexpressed human SAT1 in HEK293T cells and treated the cells with PBA. Consistent with a previous study (34), we observed a significant reduction of the acetyl-CoA level in SAT1-overexpressing cells (Figure 1B). With PBA treatment, the acetyl-CoA level was significantly recovered (Figure 1B). Another approach to assess the cellular acetyl-CoA level is by probing global protein acetylation. Using an acetylation-specific Ab, we detected a significant reduction in global protein acetylation in SAT1-overexpressing cells that could be rescued by PBA treatment (Figure 1, C and D; see complete unedited blots in the supplemental material). Notably, we observed recovered acetylation of proteins beyond histones (Figure 1D), suggesting PBA regulates protein acetylation more broadly than through its function as an HDAC inhibitor, as previously suggested (55).

Interestingly, we found that the level of overexpressed SAT1 is significantly lower in the cells with PBA treatment (Figure 1D). It has been reported that SAT1 is labile (63), and its degradation is primarily mediated by the proteasome (47). To test whether the PBA catabolite phenylacetyl-CoA could promote proteosome-mediated degradation of SAT1, we treated HEK293T cells overexpressing SAT1 with PBA, the proteasome inhibitor MG132, or the combination. As expected, MG132 treatment significantly elevated the level of the SAT1 protein (Figure 1E and unedited blots in the supplemental material). Notably, with MG132 treatment, PBA lost the activity to downregulate SAT1 (Figure 1E), suggesting PBA promotes SAT1 degradation mediated by the proteasome. As a control, PBA did not regulate the stability of EGFP (Supplemental Figure 2), and treatment with the autophagy inhibitor bafilomycin had no effect on PBA activity (Supplemental Figure 3). To further evaluate the effect of PBA on endogenous SAT1, we treated the fibroblasts of patients with SRS with N^1^,N^11^-diethylnorspermine (DENSPM) (Supplemental Figure 1), a previously characterized spermine analog and SAT1 inducer (64–68). DENSPM-induced SAT1 was significantly reduced by PBA and stabilized by MG132 (Figure 1F and unedited blots in the supplemental material). In the presence of MG132, PBA treatment had no effect on the level of SAT1 (Figure 1F), suggesting that the effect of PBA on downregulating SAT1 requires proteasome function. Glycerol-PBA (Supplemental Figure 1), an alternative form of PBA for urea cycle disorder therapy (69), regulated SAT1 protein levels in a similar way (Supplemental Figure 4). Taken together, these results indicate PBA potentially destabilizes SAT1 and suppresses SAT1-mediated acetyl-CoA reduction.

### PBA ameliorates SRS in a Drosophila model.

**Figure 2:**
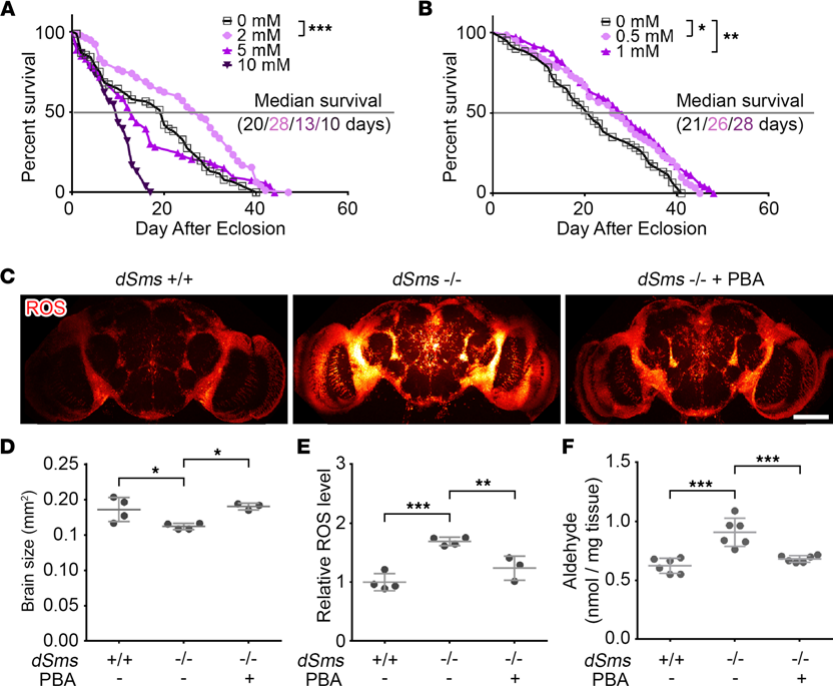
PBA treatment extends life span and reduces ROS and aldehydes in a *Drosophila* SRS model. (**A**) Life span of female SRS flies fed with indicated concentration of PBA. *n* = 76 at 0 mM, 77 at 2 mM, 53 at 5 mM, and 53 at 10 mM; log-rank (Mantel-Cox) test, Bonferroni-corrected α = 0.0167. (**B**) Life span of female SRS flies fed with lower concentration of PBA. *n* = 60, 60, and 71; log-rank (Mantel-Cox) test, Bonferroni-corrected α = 0.025. (**C**) ROS staining of brains of 10 days after eclosion (DAE) flies with or without PBA feed. Scale bar: 100 μm. The image is a representative of multiple brains in each group. (**D**) Quantification of the brain size in **C**. *n* = 4, 4, and 3. (**E**) Quantification of the relative ROS level in **C**. The ROS signal was normalized with the brain size. All the values were further normalized by that of the first WT brain. *n* = 4, 4, and 3. (**F**) Aldehyde level measurement of 10 DAE flies with or without PBA feed. Each dot indicates a sample of homogenized mixture of 10 flies. *n* = 6, 6, and 6; *P < 0.05, **P < 0.01, ***P < 0.001; ordinary 1-way ANOVA multiple comparisons in **D**–**F**. Data represent mean ± SEM.

Our previous study suggested SAT1-initiated spermidine catabolism causes toxicity in SRS (22). To test the potential therapeutic effect of PBA for SRS, we used our *Drosophila* model of SRS and treated *Drosophila Sms* (*dSms*) mutant flies with different concentrations of PBA. While 10 mM of PBA showed toxicity, 2 mM of PBA significantly extended life span (Figure 2A and Supplemental Figure 5A). Lower concentrations of 1 mM or 0.5 mM showed milder benefit (Figure 2B). In subsequent studies, we used 2 mM as the concentration for treatment. Glycerol-PBA, which integrates 3 phenylbutyrate molecules with a glycerol molecule, similarly extended the life span of SRS flies when given at 0.6 mM (Supplemental Figure 5, B and C).

Next, we examined the levels of H_2_O_2_ and aldehydes, 2 metabolites of spermidine catabolism and toxicity mediators of SRS (22). Strikingly, with the dihydroethidium (DHE) staining for ROS in the brains, we found the brains of SRS flies were significantly smaller than those of control flies, and PBA treatment significantly recovered the brain size of SRS flies (Figure 2, C and D). Consistent with our previous studies, ROS were significantly accumulated in SRS fly brains (Figure 2, C and E) and were significantly reduced by treatment with PBA (Figure 2, C and E). Similarly, aldehydes were significantly accumulated in SRS fly brains and were significantly reduced by PBA treatment (Figure 2F).

**Figure 3:**
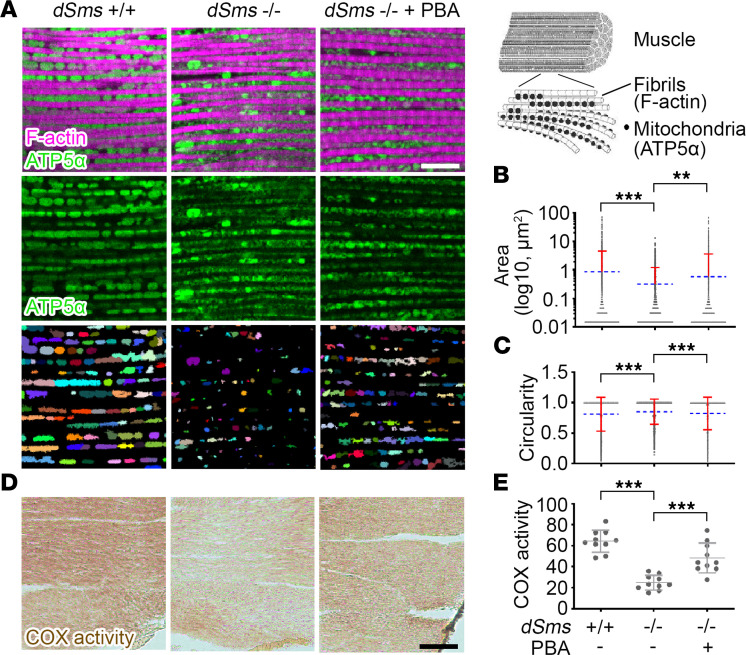
PBA treatment partially restores mitochondria in a *Drosophila* SRS model. (**A**) Mitochondrial membrane protein ATP5α and contractile filament component F-actin staining of flight muscle of 10 DAE flies with or without PBA feed. The third row shows recognized mitochondria by automatic segregation of ATP5α staining signal using ImageJ H-watershed (NIH). The images are representatives of 5 flies in each group. Scale bar: 10 μm. The diagram of muscle fiber structure on the top right is adapted from (97). (**B**) Quantification of mitochondrial size indicated by segregated ATP5α signal. Each mitochondrion is marked as a dot. Multiple dots of mitochondria with the same size aggregate into a black line. The blue dash lines indicate the average values. The red bars indicate SD. (**C**) Quantification of mitochondrial shape indicated by circularity of segregated ATP5α signal. Each mitochondrion is marked as a dot. Multiple dots of mitochondria with the same circularity aggregate into a black line. The blue dash lines indicate the average values. The red bars indicate SD. (**D**) COX activity staining of flight muscle of 10 DAE flies with or without PBA feed. The image is a representative of 10 samples in each group. Scale bar: 50 μm. (**E**) Quantification of COX activity in **D**. *n* = 10; **P < 0.01, ***P < 0.001; ordinary 1-way ANOVA multiple comparisons in **B**, **C**, and **E**. Data represent mean ± SEM.

Next, we evaluated the effect of PBA on mitochondria and lysosomes, which have been shown to be vulnerable to ROS and aldehydes, respectively, and the main defective cellular organelles in SRS (22). We examined the mitochondrial integrity using 2 approaches: i) morphological analysis by immunofluorescence labeling of mitochondrial membrane protein ATP5α for mitochondrial morphology (70) and ii) functional assessment by determining cytochrome *c* oxidase (COX) activity (71). Examining the adult flight muscle, which is homologous to the mammalian skeletal muscle (72), we found abnormal mitochondrial size and shape in the SRS fly flight muscle. PBA treatment significantly ameliorated the abnormality (Figure 3, A–C), although the general morphology of the muscle fibers in the SRS fly was similar to that in WT control flies as indicated by the contractile filament component F-actin labeling (Figure 3A), suggesting a mitochondria-specific phenotype in SRS. To further assess the effect of PBA treatment on mitochondrial function, we measured COX activity and found a significant reduction in the SRS fly flight muscle (Figure 3, D and E) and a remarkable restoration with PBA treatment (Figure 3, D and E).

**Figure 4:**
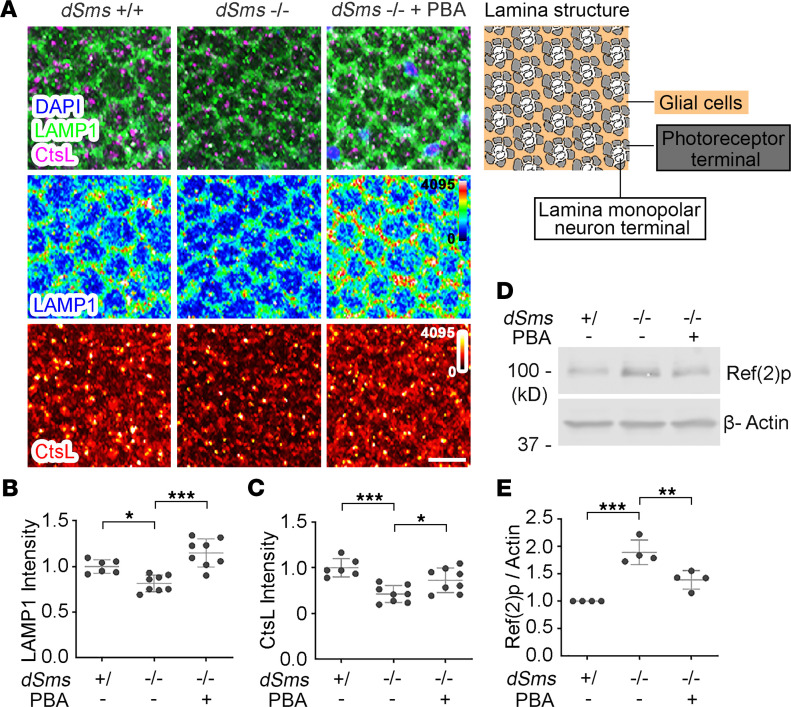
PBA treatment improves lysosomal function in a *Drosophila* SRS model. (**A**) Lysosomal membrane protein LAMP1 and CtsL staining of lamina of 10 DAE flies with or without PBA feed. The diagram of lamina structure is on the top right. Scale bar: 10 μm. (**B**) Quantification of LAMP1 or (**C**) CtsL intensity in **A**. *n* = 6, 6, and 8; ordinary 1-way ANOVA multiple comparisons. (**D**) Western blot of autophagy cargo recruiter Ref(2)p in heads of 10 DAE flies with or without PBA feed. The image is a representative of 4 separate experiments. (**E**) Quantification of the Ref(2)p level in **D**. The Ref(2)p level was normalized with the β-Actin level. All the values were further normalized by that of WT flies. *n* = 4; *P < 0.05, **P < 0.01, ***P < 0.001; 1-way ANOVA multiple comparisons (matched). Data represent mean ± SEM.

To examine the integrity of lysosomes, we probed for the lysosomal membrane protein 1 (LAMP1) and the lysosomal proteinase cathepsin L (CtsL) in lamina synapses. Consistent with our previous observation (22), both LAMP1 and CtsL levels were significantly downregulated in SRS flies (Figure 4). However, with PBA treatment, both LAMP1 and CtsL levels were significantly rescued (Figure 4). Furthermore, we evaluated the functional autolysosome flux indicated by the Refractory to Sigma P (Ref(2)p) protein level. Ref(2)p, the homolog of human p62/Sequestosome 1 (SQSTM1), is the recruiter of autolysosome cargo, which is degraded together with the cargo (73). Blocked autolysosome flux causes both cargo and cargo recruiter accumulation (73). Consistent with our previous studies (22), we detected elevated levels of Ref(2)p in SRS fly heads (Figure 4, D and E, and unedited blots in the supplemental material). With PBA treatment, the Ref(2)p level was significantly reduced (Figure 4, D and E), suggesting the recovery of autophagic flux.

### PBA improves lysosomal function in fibroblasts of patients with SRS.

**Figure 5:**
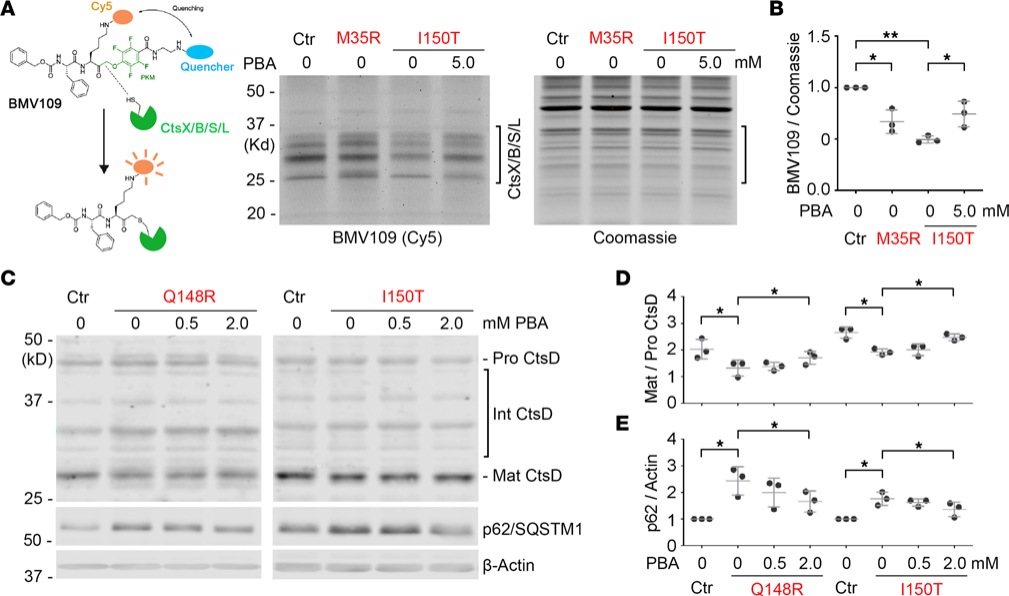
PBA treatment improves lysosomal function in fibroblasts of patients with SRS. (**A**) BMV109(Cy5) labeling of active lysosomal Cts in patient fibroblasts (Ctr, GM09503; M35R, CMS25081a; and I150T, CMS1849b) with indicated SMS mutation with or without PBA treatment. The image is a representative of 3 separate experiments. (**B**) Quantification of the BMV109(Cy5) intensity of the strongest band in the bracket area, normalized with Coomassie staining signal in the bracket area in **A**. All the values were further normalized by that of the control cells. (**C**) Western blot of CtsD and autophagy cargo recruiter p62/SQSTM1 in patient fibroblasts (Ctr, CMS24833a; Q148R, CMS4627; and I150T, CMS1849a) with indicated SMS mutation with or without PBA treatment. The image is a representative of 3 separate experiments. (**D**) Quantification of the ratio of matured CtsD to pro CtsD in **C**. (**E**) Quantification of the p62/SQSTM1 level in **C**. The p62/SQSTM1 level was normalized with the β-Actin level. All the values were further normalized by that of the control cells. In **B**, **D**, and **E**, *n* = 3; *P < 0.05, **P < 0.01; 1-way ANOVA multiple comparisons (matched). Data represent mean ± SEM.

To further assess the therapeutic potential of PBA in treating SRS, we evaluated the effect of PBA on the cellular phenotypes of fibroblasts of patients with SRS, specifically the lysosomal defects in SRS (22). First, we used an activity-based probe for cysteine cathepsins (Cts), BMV109, to detect active Cts, which are activated in functional lysosomes (Figure 5A and Supplemental Figure 6) (74). Active Cts X/B/S/L were significantly reduced in fibroblasts of patients with SRS, compared with those in control fibroblasts (Figure 5, A and B, and unedited blots in the supplemental material). With PBA treatment, the levels of active Cts in patient fibroblasts were significantly restored (Figure 5, A and B). Next, we examined the maturation of aspartyl lysosomal proteinase cathepsin D (CtsD). Consistent with our previous studies (22), the maturation of CtsD, as indicated by the ratio of matured CtsD to pro CtsD, was significantly decreased in patient fibroblasts (Figure 5, C and D, and unedited blots in the supplemental material). With PBA treatment, the maturation of CtsD was significantly recovered in patient fibroblasts (Figure 5, C and D). Furthermore, we evaluated functional autophagic flux as indicated by protein level of the cargo recruiter p62/SQSTM1. Consistent with our observation in flies (22), we detected elevated levels of p62/SQSTM1 in fibroblasts of patients with SRS (Figure 5, C and E, and unedited blots in the supplemental material). With PBA treatment, the p62/SQSTM1 level was significantly reduced (Figure 5, C and E), suggesting the recovery of autophagic flux in fibroblasts of patients with SRS. We also probed for LC3B-I/-II proteins in these cells but did not observe a significant difference between control cells and patient cells (data not shown). The observations using these 2 markers suggest that in the cells of patients with SRS, the lysosomes, not the phagophores, are the most impacted step in the autophagic flux.

### PBA restores both cellular acetyl-CoA level and protein acetylation reduction in SRS.

**Figure 6:**
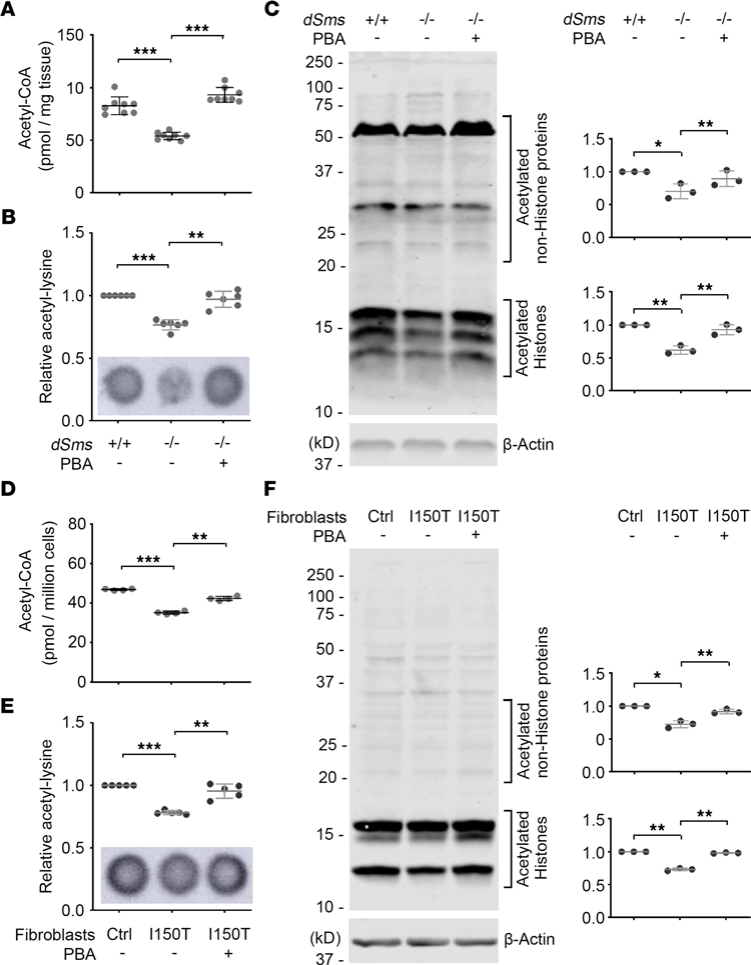
PBA treatment restores acetyl-CoA and protein acetylation in a *Drosophila* SRS model and fibroblasts of patients with SRS. (**A**) Acetyl-CoA level in 10 DAE flies with or without PBA feed. Each dot indicates a sample of homogenized mixture of 10 flies. *n* = 8; ordinary 1-way ANOVA multiple comparisons. (**B**) Dot blot of acetyl-lysine in 10 DAE flies with or without PBA feed. The values of the quantification were normalized with the WT fly samples. Each dot indicates a sample of homogenized mixture of 10 flies. *n* = 6. (**C**) Western blot of acetyl-lysine in 10 DAE flies with or without PBA feed. Acetylated histones and nonhistone proteins in the bracket areas were quantified separately and normalized with the β-Actin level. All the values were further normalized by that of the WT flies. *n* = 3. (**D**) Acetyl-CoA level in patient fibroblasts (Ctrl, CMS24833a; I150T, CMS1849a) with or without PBA treatment. Cells from 4 separate experiments were stored at –80°C and then tested in a single plate. *n* = 4. (**E**) Dot blot of acetyl-lysine in patient fibroblasts with or without PBA treatment. The values of the quantification were normalized with the control cells. *n* = 5. (**F**) Western blot of acetyl-lysine in patient fibroblasts with or without PBA treatment. Acetylated histones and nonhistone proteins in the bracket areas were quantified separately and normalized with the β-Actin level. All the values were further normalized by that of the control cells. *n* = 3; *P < 0.05, **P < 0.01, ***P < 0.001; 1-way ANOVA multiple comparisons (matched) in **B**–**F**. Data represent mean ± SEM.

The beneficial effect of PBA on the *Drosophila* model of SRS and on patient fibroblasts is highly promising. To uncover the mechanism of drug action and determine the targeting specificity of PBA on SAT1 in vivo, we measured the level of acetyl-CoA, the substrate of SAT1-mediated spermidine acetylation, in SRS flies with or without PBA treatment. Strikingly, we found that the acetyl-CoA level in SRS flies was significantly reduced compared with that of control flies, and PBA treatment significantly recovered the acetyl-CoA level of SRS flies (Figure 6A). Consequently, protein acetylation was significantly reduced in SRS flies, and the reduction was ameliorated by PBA treatment (Figure 6, B and C, and unedited blots in the supplemental material). As in SAT1-overexpressing cells, PBA recovered acetylation of proteins beyond histones in SRS flies (Figure 6C), suggesting PBA potentially regulates global protein acetylation through modulating acetyl-CoA level in vivo, acting beyond just an HDAC inhibitor as previously indicated (55). A similar effect of PBA on acetyl-CoA and protein acetylation was observed in fibroblasts of patients with SRS (Figure 6, D–F, and unedited blots in the supplemental material). We further validated the alteration of protein acetylation by staining the fibroblasts with an acetyl-lysine Ab. Strong acetyl-lysine staining was observed to be highly enriched in the nucleus, consistent with the observation in Western blot analysis where histone bands were the main targets of acetylation. Nuclear acetyl-lysine signal was significantly reduced in fibroblasts of patients with SRS, and PBA treatment significantly increased the acetyl-lysine detected (Supplemental Figure 7).

### PBA exerts heterogeneous effects on polyamine levels.

**Figure 7:**
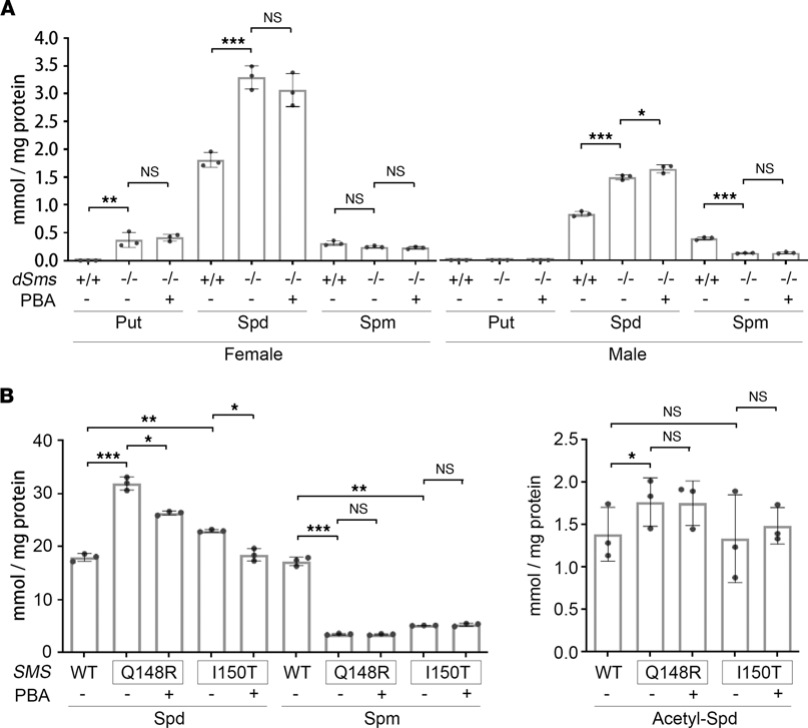
PBA heterogeneously regulates polyamine level. (**A**) PBA treatment does not restore the polyamine levels in SRS flies. No acetyl-spermidine was detected in any of the samples. Each dot indicates a sample of homogenized mixture of 10 flies. *n* = 3; ordinary 1-way ANOVA multiple comparisons. (**B**) PBA treatment regulates spermidine level in fibroblasts of patients with SRS. No putrescine was detected in any of the samples. Each dot indicates an individual drug treatment experiment. *n* = 3; *P < 0.05, **P < 0.01, ***P < 0.001; 1-way ANOVA multiple comparisons (matched). Data represent mean ± SEM. Put, putrescine; Spd, spermidine; Spm, spermine.

The effect of PBA on SAT1 and acetyl-CoA levels would predict an inhibition of polyamine acetylation and catabolism and a possible change in polyamine level. We first determined the levels of polyamines in the flies. Consistent with our previous studies (22), spermidine was significantly accumulated in SRS flies (Figure 7A). However, to our surprise, no significant change in the polyamine levels was observed in PBA-treated groups (Figure 7A), suggesting that modulating SAT1 and the cellular acetyl-CoA level had a modest impact on the polyamine steady state level in vivo in flies. We then measured the levels of polyamines in fibroblasts of patients with SRS. Consistent with our previous studies (75), spermidine was significantly accumulated and spermine was significantly reduced in fibroblasts of patients with SRS, although the levels in different patient cell lines varied (Figure 7B). Interestingly, PBA treatment significantly reduced the spermidine levels in fibroblasts of patients with SRS who carry either the Q148R or I150T mutation to 17.4% ± 2.4% and 19.7% ± 5.8%, respectively (Figure 7B), suggesting that spermidine accumulation in SRS fibroblasts was sensitive to PBA treatment. Furthermore, we examined the level of N^1^-acetyl-spermidine in patient fibroblasts and detected a higher level of N^1^-acetyl-spermidine in Q148R fibroblasts than in I150T fibroblasts, consistent with its higher spermidine level (Figure 7B). However, PBA treatment showed no significant effect on the acetyl-spermidine level in either cell line (Figure 7B). Taken together, PBA treatment significantly reduced spermidine accumulation in fibroblast cells of patients with SRS, although the steady-state level of spermine and N^1^-acetyl-spermidine were not altered (Figure 7B).

## Discussion

Polyamine metabolism is a key process for cell growth and stress response. The dysregulation of polyamine metabolism causes multisystem, syndromic disorder (19–21). SRS is the first genetic syndrome associated with the polyamine metabolic pathway (19). The multisystem manifestation of SRS underscores the systemic consequences of dysregulation of polyamine metabolism. Our previous studies revealed ROS and aldehyde accumulation impairing mitochondria and lysosome as key factors in SRS pathogenicity. In this study, we discovered that acetyl-CoA reduction and altered protein acetylation are potentially novel pathomechanisms of SRS. We proposed to repurpose the FDA-approved drug PBA to treat SRS by attenuating the spermidine acetylase SAT1 and restoring cellular acetyl-CoA levels.

Polyamine levels are tightly regulated at the level of synthesis, catabolism, and uptake from and excretion to extracellular environments (1). Under SRS conditions, SMS deficiency blocks spermine synthesis from spermidine, resulting in spermidine accumulation (Figure 7) (21, 22, 75, 76). The level of substrates for polyamine synthesis and byproducts of catabolism is also significantly affected by dysregulated polyamine metabolism (1, 2). Increased polyamine synthesis consumes more S-adenosyl methionine, while increased catabolism consumes more acetyl-CoA and produces more ROS and aldehydes (1, 2, 77). In this study, we showed that, in addition to the accumulation of toxic ROS and aldehydes, acetyl-CoA reduction is also a phenotype in these models of SRS (Figure 2 and Figure 6).

In addition to SAT1-mediated polyamine acetylation, acetylation of many other small molecules and proteins, such as choline and histones, also use acetyl-CoA as the acetyl group donor (78, 79). Fluctuation of acetyl-CoA concentration or acetyl-CoA/CoA ratio significantly regulates the acetylation of these substrates (78, 79). For example, thiamine deficiency causes a decrease of synaptoplasmic acetyl-CoA levels, resulting in a reduction of acetylcholine release in synaptic terminals (80). Nutrient depletion–caused decrease of cytosolic acetyl-CoA levels reduces activity of the acetyltransferase E1A binding protein P300 to activate autophagy (81). Downregulation of acyl-coenzyme A synthetase short-chain family member 2 causes a decrease of nuclear acetyl-CoA levels that reduces histone acetylation and responsive gene expression (82, 83). Our observation that PBA treatment regulates cellular the acetyl-CoA level would indicate a potentially global impact of PBA on all the cellular pathways that depend on acetyl-CoA.

PBA and its derivative phenylacetate have been extensively studied as aliphatic acid-based HDAC inhibitors (84), especially in the context of cancer therapy (55). PBA has been shown to rebalance histone acetylation to inhibit proliferation, induce differentiation, and promote cell cycle arrest or apoptosis of different types of cancer cells (85–88). However, the cellular response to PBA is highly variable, and the clinical trials of PBA on cancer therapy have not yet been successful (86, 89, 90). Our studies add the acetyl-CoA level as another cellular target of PBA to rebalance acetylation of proteins, including histones and other nonhistone proteins. This discovered effect of PBA on acetyl-CoA level restoration might explain the complexity of cellular responses as well as the disappointing outcomes of PBA clinical trials. Specifically, it is likely that, on the one hand, PBA inhibits tumor progression in experimental cancer therapeutics through inhibiting HDAC activity, and on the other hand, PBA restores the cellular acetyl-CoA level and potentially promotes cancer cell survival in the nutrient-deprived tumor microenvironment. This would suggest the potential beneficial effect of combining PBA with inhibitors of acetyl-CoA production in treating tumors.

PBA treatment potentially downregulates SAT1 and inhibits polyamine acetylation, the first and the rate-limiting step of polyamine catabolism (Figure 1). Surprisingly, while the polyamine catabolism substrate acetyl-CoA and the byproducts H_2_O_2_ and aldehydes were significantly altered, the absolute levels of polyamines in SRS flies were not significantly restored by PBA treatment (Figure 7A). Interestingly, in fibroblasts of patients with SRS, the spermidine accumulation was reduced by PBA treatment (Figure 7B). The observed difference in the effect of PBA on the spermidine level between fibroblast cultures of patients with SRS and flies in vivo might result from possible cell type-specific responses to PBA as a SAT1 modulator or HDAC inhibitor (91). While fibroblasts of patients with SRS are sensitive to PBA treatment, other cell types may be less sensitive. It is likely that when the whole fly brain or body was homogenized and extracted for polyamine measurement, the change in the polyamine level in response to PBA treatment might be below the detection limit.

Although SAT1 plays important roles in many different physiological or pathological conditions, endogenous SAT1 protein level is very low, below the detection limit of Western blotting (35, 36, 39, 40, 42, 92). Therefore, monitoring the SAT1 protein level or activity in *Drosophila* or cell lines is challenging. Although it has been shown that SAT1 activity is not significantly altered in SRS cell lines (75), whether SAT1 activity in vivo in specific tissues is dysregulated under SRS remains to be determined. SAT1 protein is labile, with a half-life of about 30 minutes in cells (47, 63). We attempted to measure the decay curve of overexpressed or DENSPM-induced SAT1 with or without PBA treatment but obtained inconsistent results. An in vitro cell-free lysate system might be more feasible in determining the degradation rate. Such an in vitro system might also be useful to analyze the competitive effect of PBA-derived phenyl-acetyl-CoA on acetyl-CoA.

In addition to acetylation of its N^1^ position by SAT1, spermidine can be modified by a yet to be definitively determined acetyltransferase to N^8^-acetyl-spermidine (93, 94), which can be deacetylated by HDAC10 (95). N^8^-acetylspermidine has been reported to have increased in the plasma of 3 SRS patients (76). However, despite numerous attempts, we were unable to detect N^8^-acetylspermidine in SRS flies or in patient lymphocytes (75). Whether the spermidine N^8^-acetyltransferase and the corresponding deacetylase HDAC10 play any role in specific tissues in SRS remains to be determined. Further studies of PBA on N^8^-acetylation and deacetylation of spermidine would be warranted upon the confirmation of the cellular presence of N^8^-acetylspermidine and the identification of the acetyltransferase that catalyzes N^8^-acetylation.

In conclusion, we identified altered cellular acetyl-CoA level and protein acetylation as potentially novel pathomechanisms of SRS and revealed the therapeutic potential for PBA in treating SRS. Our findings of the effects of PBA on SAT1, acetyl-CoA and protein acetylation shed light on the potential of PBA as a therapeutic agent for other polyamine metabolism-related diseases.

## Methods

### Cell culture and drug administration.

HEK293T cells (ATCC, catalog CRL-3216) were cultured in Advanced DMEM/F12 medium (Gibco, catalog 12634-010) supplemented with 5% FBS (ATCC, catalog 30-2020) at 37°C with 5% CO_2_ in the VWR symphony incubator. For drug treatment, drugs were added into the cell culture medium in a tube, mixed by pipetting, and then transferred into wells or dishes with cells.

Fibroblast cell lines (marked with *SMS* genotype and patient number) established from skin biopsies of patients with SRS or healthy donors were transferred from Greenwood Genetic Center and cultured in RPMI-1640 Medium (MilliporeSigma, catalog R0883) supplemented with 15% FBS (ATCC, catalog 30-2020) at 37°C with 5% CO_2_ in the VWR symphony incubator. For DENSPM, PBA, and proteasome inhibitor MG132 treatment, the cells were incubated in medium with DENSPM (100 μM) for 18 hours and then incubated in fresh medium with PBA or MG132 (10 μM) for 2 hours.

### Plasmid and transfection.

Plasmid pCMV-HA-SAT1was constructed by inserting the human SAT1 coding sequence (CDS) into vector pCMV-HA. The SAT1 CDS was amplified from Addgene plasmid 25493 (a gift from Cheryl Arrowsmith, University Health Network, Toronto, Ontario, Canada). For transfection, the plasmid was mixed with jetPRIME transfection reagent (Polyplus, catalog 114-07) according to the instructions from the manufacturer. HEK293T cells were detached with 0.05% Trypsin-EDTA (Gibco), resuspended with cell culture medium, mixed with plasmid transfection reagent mixture, and then transferred into wells or dishes.

### Drosophila culture and drug administration.

*dSms* mutant fly strain has been previously characterized (22). Flies were maintained on a cornmeal-molasses-yeast medium at 22°C, 65% humidity, and 12 hours light/12 hours dark. For drug treatment, newly eclosed adults were transferred to vials containing food supplemented with the indicated concentration of drugs. The food was changed every week.

### Fly life span assay.

Newly eclosed flies were collected, and about 20 flies of the same sex were kept in a fresh food vial with or without the indicated concentration of drugs. Flies were transferred to new vials every week, and the number of live flies was counted every other day.

### ROS detection and quantification.

ROS detection was performed as described with minor adjustments (22). Briefly, 1 mg of DHE was dissolved in 100 μL of DMSO and then diluted to 30 μM with Schneider’s medium (SM) right before use. Flies were dissected in SM at room temperature, and the brains were incubated in DHE solution in the dark for 15 minutes. After washing with PBS for 5 minutes, the brains were mounted on glass slides with VECTASHIELD Antifade Mounting Medium (Vector Laboratories). Imaging was performed within 4 hours. Quantification was carried out using ImageJ software.

### Aldehyde measurement.

A total of 10 flies were weighed and homogenized in assay buffer (MilliporeSigma, catalog MAK141; 100 μL per 5 mg of tissue). The samples were centrifuged at 13,523 × *g* at room temperature for 1 minute. Subsequent steps were done according to the instructions from the kit manufacturer (MilliporeSigma, catalog MAK141).

### Fly flight muscle dissection and IHC staining.

Flight muscles were dissected in cold PBS and fixed in freshly made 4% formaldehyde for 15 minutes. After 10 minutes washing in PBS containing 0.4% (v/v) Triton X-100 (PBTX) 3 times, the muscles were incubated with ATP5α Abs diluted in 0.4% PBTX containing 5% goat serum overnight at 4°C. The muscles were then incubated at room temperature with conjugated secondary Abs for 2 hours, followed by staining with DAPI for 10 minutes. After washing, the muscles were mounted on glass slides with VECTASHIELD Antifade Mounting Medium (Vector Laboratories) and kept at 4°C until imaging. Specimens were imaged using an Olympus IX81 confocal microscope. Images were processed using FluoView 10-ASW software (Olympus) and analyzed using ImageJ software.

### COX activity staining.

COX activity was stained as described (71). Briefly, 10 μm fresh ultra-rapid frozen flight muscle sections were collected and stored at −20°C until ready to use. Samples were dried at room temperature for 15 minutes, and then incubated in 20 μL of staining solution (1 mg/mL 3,3′-diaminobenzidine [DAB], 1 mg/mL cytochrome *c*, and 2 μg/mL catalase in 5 mM PBS) for 20 minutes at room temperature and washed with PBS for 10 minutes 4 times. The samples were then dehydrated for 2 minutes in each of the following concentrations of ethanol: 70%, 70%, 95%, 95%, and 99.5%, followed by an additional 10 minutes of dehydration in 99.5% ethanol. The slides were then placed in xylene for 10 minutes, mounted with Entellan Mounting Medium and coverslips, and allowed to dry overnight before imaging. Quantification was carried out using ImageJ software. COX activity was indicated by mean gray value of DAB signals.

### Fly brain dissection and IHC staining.

Brains with attached lamina were dissected in cold PBS and fixed in freshly made 4% formaldehyde for 15 minutes. After 10 minutes washing in PBS containing 0.4% (v/v) Triton X-100 (PBTX) 3 times, brains were incubated with primary Abs diluted in 0.4% PBTX containing 5% goat serum overnight at 4°C. Brains were then incubated at room temperature with conjugated secondary Abs for 2 hours, followed by staining with DAPI for 10 minutes. After washing, brains were mounted on glass slides with VECTASHIELD Antifade Mounting Medium (Vector Laboratories) and kept at 4°C until imaging. Specimens were imaged using an Olympus IX81 confocal microscope. Images were processed using FluoView 10-ASW software (Olympus) and analyzed using ImageJ software.

### Abs and reagents.

The following commercially available Abs were used: anti–acetyl-lysine (Cell Signaling Technology, catalog 9441s), anti-SAT1 (Cell Signaling Technology, catalog 61586s), anti-ATP5α (Abcam, catalog Ab14748), anti-Drosophila LAMP1 (Abcam, catalog ab30687), anti–cathepsin L (R&D Systems, catalog MAB 22591), anti-Ref(2)p (Abcam, catalog ab178440), anti–cathepsin D (Cell Signaling Technology, catalog2284), anti-p62/SQSTM1 (Novus Biologicals, catalog NBP1-48320), and secondary Abs conjugated to Alexa Fluor 488/555/647 (Thermo Fisher Scientific, catalog A11001/A21422/A21235), or Cy3/Cy5 (Rockland, catalog 611-110-122/611-104-122), or near-infrared dye 700/800 (Rockland, catalog 611-144-102/610-145-002). The following chemicals were used in the study: sodium phenylbutyrate (MilliporeSigma, catalog SML0309), glycerol phenylbutyrate (Molport, catalog cs-0017499), MG132 (MilliporeSigma, catalog M8699), N1,N11-diethylnorspermine (Santa Cruz Biotechnology, catalog sc-204114), DHE (Thermo Fisher Scientific, catalog D11347), and phalloidin conjugated with Alexa Fluor 546 (Invitrogen, catalog A22283).

### Immunoblot analysis.

For fly acetyl-lysine detection, fly whole bodies were homogenized in RIPA buffer (Thermo Fisher Scientific, catalog R0278) containing cOmplete protease inhibitor cocktail (Roche, catalog 11836170001), followed by centrifugation at 12,000 × *g* at 4°C for 5 minutes. For fly head Ref(2)p detection, fly heads were homogenized in RIPA buffer containing cOmplete protease inhibitor cocktail, followed by centrifugation at 12,000 × *g* at 4°C for 5 minutes. For cellular acetyl-lysine detection, cells were lysed with RIPA buffer containing cOmplete protease inhibitor cocktail on ice for 15 minutes, followed by centrifugation at 12,000 × *g* at 4°C for 5 minutes. For cellular SAT1 or HA-SAT1 detection, cells were lysed with M-PER buffer (Thermo Fisher Scientific, catalog 78503) containing cOmplete protease inhibitor cocktail on ice for 10 minutes, followed by centrifugation at 12,000 × *g* at 4°C for 5 minutes. For Western blot analysis, the supernatants were mixed with Laemmli sample buffer and heated at 95°C for 10 minutes. Proteins were separated on a Bis-Tris 10%–15% gel and transferred to a nitrocellulose membrane. For dot blot analysis, the supernatants were directly dotted onto a nitrocellulose membrane and dried for 30 minutes. After blocking, the membrane was incubated with primary Abs overnight at 4°C and then with near-infrared dye–conjugated secondary Abs for 2 hours at room temperature. Imaging was carried out on an Odyssey Infrared Imaging system (LI-COR Biosciences), and images were analyzed using Image Studio software.

### Acetyl-CoA measurement.

Acetyl-CoA was measured with a fluorometric assay kit (MilliporeSigma, catalog MAK039) following the instructions from the manufacturer with minor adjustments. Briefly, 5 flies or 10 million HEK293T cells or fibroblasts, stored at –80°C, were homogenized in 100 μL of 1 M perchloric acid, and 20 μL of neutralized samples was used for the measurement.

### Cts activity assay.

WT or patient fibroblast cells were labeled with 40 μL (96-well dish) or 300 μL (12-well dish) of BMV109 (50 nM) in DMEM in a CO_2_ incubator for 1 hour. For BMV109 labeling with PBA treatment, cells were preincubated with PBA for 3 days before BMV109 labeling. For BMV109 labeling with E64d, cells were pretreated with 10 μM of E64d for 30 minutes and then treated with media containing both 10 μM of E64d and BMV109. After 1 hour of labeling with BMV109, cells were washed with DPBS and lysed by scraping on plate with 100 mM citrate buffer, pH 5.5 containing 0.5% CHAPS, 0.75% Triton X-100, and protease inhibitor cocktail. The collected lysates in Eppendorf tubes were vigorously vortexed and incubated on ice for 30 minutes followed by centrifugation (20,000 × *g*). The supernatants were resolved in 12% SDS-PAGE, and then the gel was washed in deionized water for 5 minutes 3 times before imaging with ChemiDoc imager (Cy5). After imaging for BMV109-labeled cathepsins, the gel was stained with Coomassie to confirm equal protein loading.

### Polyamine measurement.

Polyamine content was determined by the precolumn dansylation, high-performance liquid chromatography method of Kabra et al. using 1,7-diaminoheptane as the internal standard (96).

### Statistics.

Data were analyzed with Prism (GraphPad Software). Log-rank (Mantel-Cox) test with multiple comparisons correction (Bonferroni method) was used for survival curve (life span) analysis. The 1-way ANOVA multiple comparisons was used for other assays. A *P* value smaller than 0.05 is considered statistically significant. *P < 0.05, **P < 0.01, and ***P < 0.001.

### Study approval.

Human fibroblast collecting and handling for all experiments complied with the policies of the Greenwood Genetic Center (GGC). Patients provided consent to the research under an approved IRB protocol to the GGC (Self Regional Healthcare; Pro00085001).

## Author contributions

XT, YZ, RS, and RGZ conceptualized the study. XT, YZ, ZDP, SHY, JRF, TMS, RAC, RS, and RGZ designed the study’s methodology. XT, YZ, ZDP, SHY, JRF, TMS, RACJ, RS, and RGZ conducted the investigation. XT, YZ, and RGZ conducted the visualizations. RS and RGZ acquired funding for the study. RGZ administered and supervised the project. XT and RGZ wrote the original draft, and YZ, TMS, RACJ, and RS reviewed and edited the manuscript.

## Supplementary Material

Supplemental data

## Version 1 07/08/2022

Electronic publication
