# Supplementary material for: Phenylbutyrate modulates polyamine acetylase and ameliorates Snyder-Robinson syndrome in a Drosophila model and patient cells
Source: JCI Insight. 2022 Jul 8;7(13):e158457. doi: 10.1172/jci.insight.158457 (PMC9310527; doi:10.1172/jci.insight.158457)
Supplement: Supplemental data [file jciinsight-7-158457-s057.pdf]

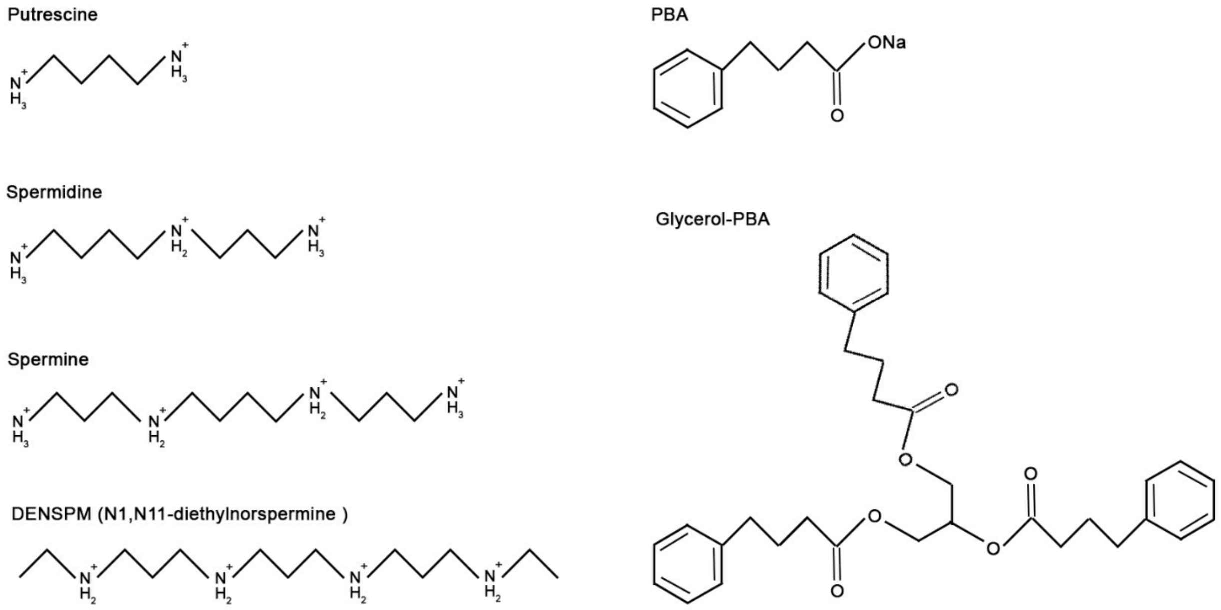

Supplemental Figure 1. Diagram of structures of PBA, polyamines and DENSPM.

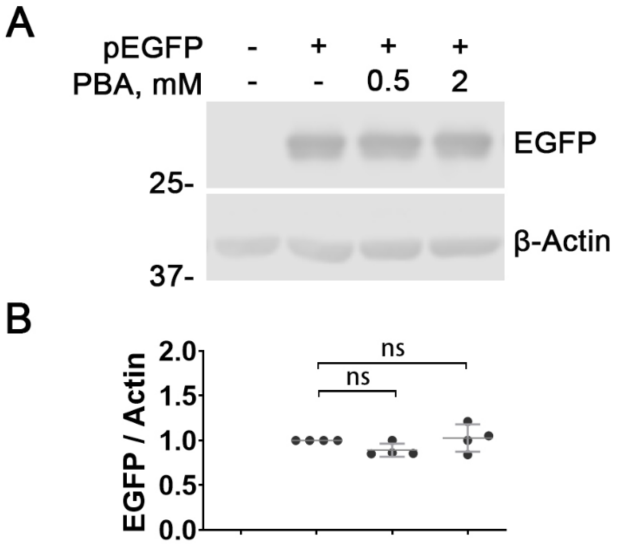

Supplemental Figure 2. PBA does not regulate EGFP stability. (**A**) Western-blot of EGFP overexpressed in HEK293T cells with indicated concentration of PBA treatment. The image is a representative of four separate experiments. (**B**) Quantification of EGFP in (**A**). The EGFP level was normalized with the β-Actin level. The values of the cells without EGFP plasmid transfection were set as background. All the values were further normalized by that of the cells with EGFP plasmid transfection but not PBA treatment. n = 4; one-way ANOVA multiple comparisons (matched). Data represent mean ± SEM.

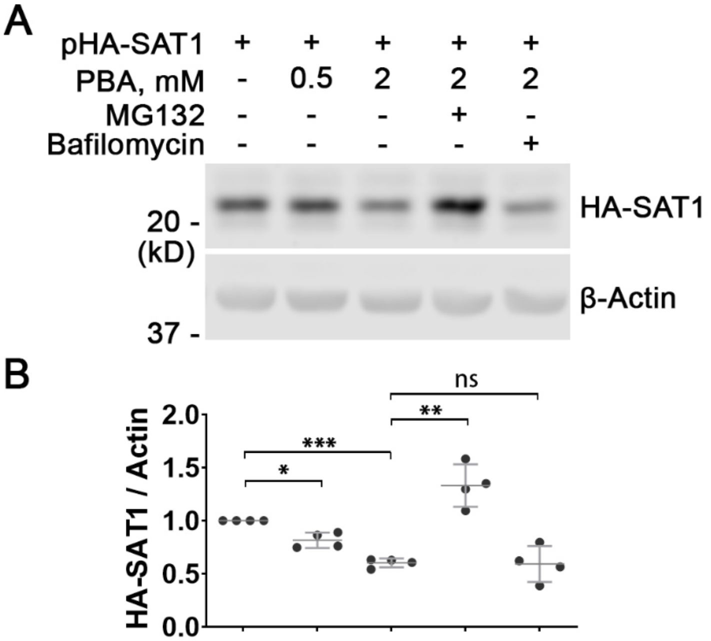

Supplemental Figure 3. Bafilomycin does not affect SAT1 with PBA treatment. (**A**) Western-blot of HA-SSA1 overexpressed in HEK293T cells with PBA or PBA plus MG132 or Bafilomycin treatment. The image is a representative of four separate experiments. (**B)** Quantification of HA-SAT1 in (**A**). The HA-SAT1 level was normalized with the β-Actin level. All the values were further normalized by that of the cells with HA-SAT1 plasmid transfection but not drug treatment. n = 4; one-way ANOVA multiple comparisons (matched). Data represent mean ± SEM.

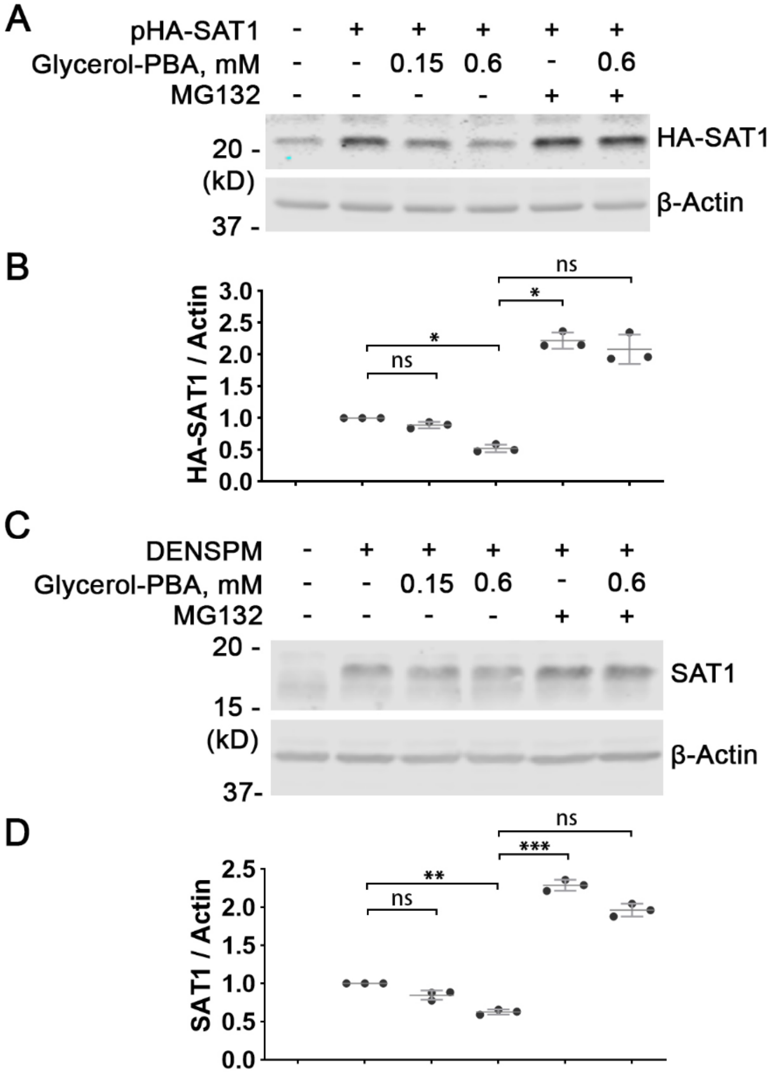

Supplemental Figure 4. Glycerol-PBA attenuates SAT1. (**A**) Western-blot of HA-SAT1 overexpressed in HEK293T cells with Glycerol-PBA, MG132 or the combination treatment. The image is a representative of three separate experiments. (**B**) Quantification of HA-SAT1 in (**A**). The HA-SAT1 level was normalized with the β-Actin level. The values of the cells without HA-SAT1 plasmid transfection were set as background. All the values were further normalized by that of the cells with HA-SAT1 plasmid transfection but not Glycerol-PBA or MG132 treatment. (**C**) Western-blot of SAT1 induced by DENSPM in patient fibroblasts (I150T, CMS1849a) with PBA, MG132 or the combination treatment. The image is a representative of three separate experiments. (**D**) Quantification of SAT1 in (**C**). The SAT1 level was normalized with the β-Actin level. The values of the cells without DENSPM treatment were set as background. All the values were further normalized by that of the cells with DENSPM but not Glycerol-PBA or MG132 treatment. (**B**, **D**) n = 3; one-way ANOVA multiple comparisons (matched). Data represent mean ± SEM.

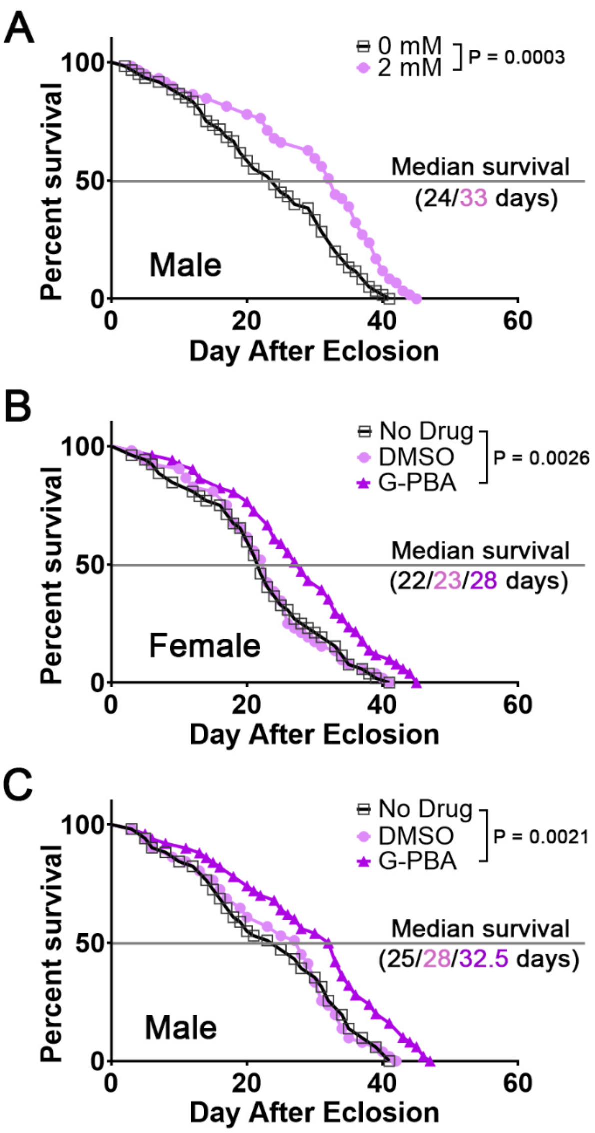

Supplemental Figure 5. PBA or Glycerol-PBA treatment extends lifespan of SRS flies. (**A**) Lifespan of male SRS flies fed with indicated concentration of PBA. n = 60, 59; Log-rank (Mantel-Cox) test. (**B**) Lifespan of female SRS flies fed with vehicle (0.1% DMSO) or 0.6 mM Glycerol-PBA. n = 52, 52, 51; Log-rank (Mantel-Cox) test, Bonferroni-corrected α = 0.025. (**C**) Lifespan of female SRS flies fed with vehicle (0.1% DMSO) or 0.6 mM Glycerol-PBA. n = 51, 51, 50; Log-rank (Mantel-Cox) test, Bonferroni-corrected α = 0.025.

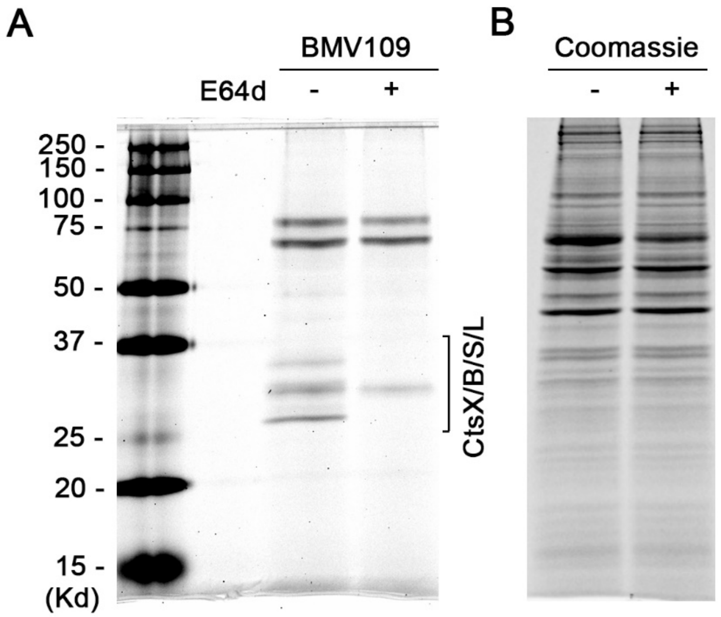

Supplemental Figure 6. Validation of BMV109 labeling of active cysteine cathepsin proteases in fibroblasts. (**A**) Wildtype fibroblasts were incubated with 50 nM of BMV109, an activity-based probe for cysteine cathepsin proteases, in the presence or absence of the pan-cysteine cathepsin inhibitor E64d. Cy5 signal indicates active cysteine cathepsins. (**B**) Coomassie staining indicates total proteins on the same membrane of (**A**).

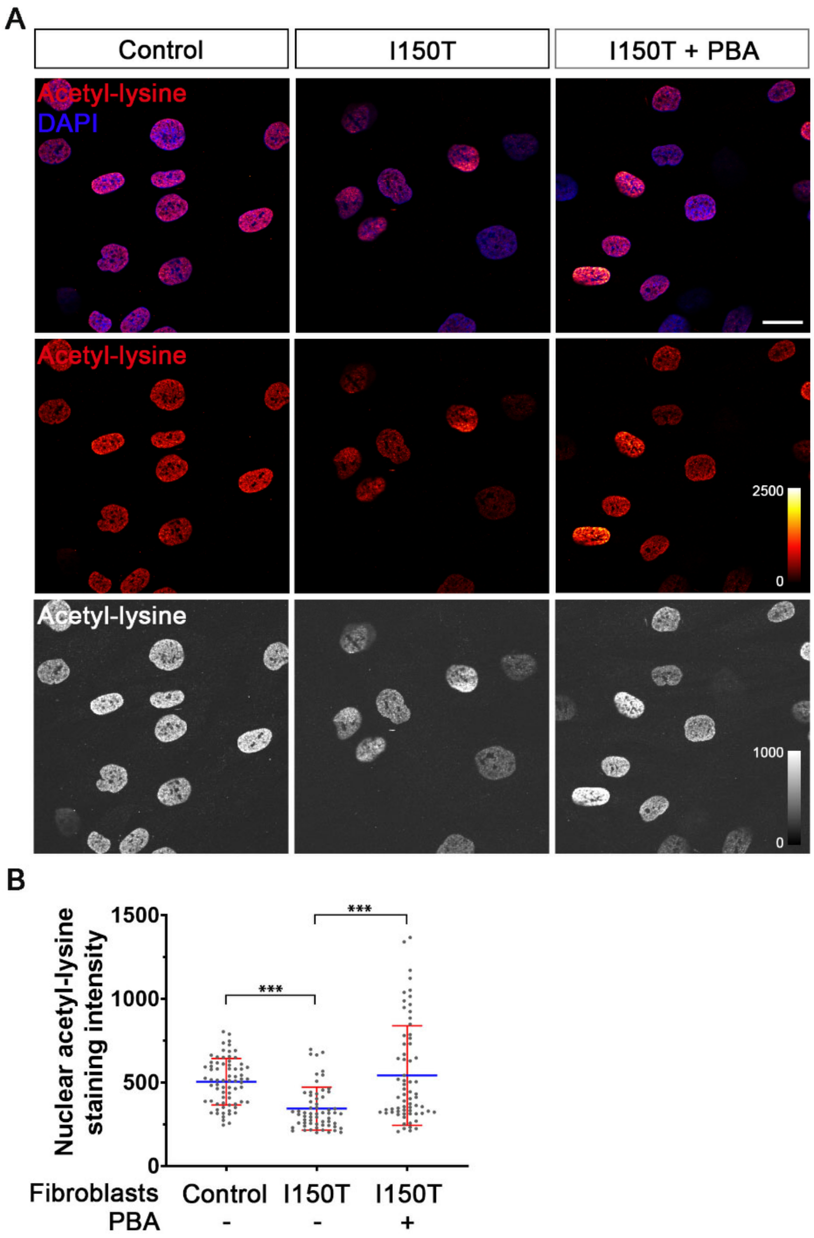

Supplemental Figure 7. PBA treatment restores protein acetylation in SRS patient fibroblasts. (**A**) Protein acetylation staining of patient fibroblasts (Ctrl, CMS24833a; I150T, CMS1849a) with or without PBA treatment using the acetyl-lysine antibody. The image is a representative of multiple fields in each group. Scale bar, 20 µm. (**B**) Quantification of nuclear acetyl-lysine staining signal intensity (A). Each dot indicates a nucleus. The blue lines indicate the average values. The red bars indicate STDEV. n = 71, 61, 67; ordinary one-way ANOVA multiple comparisons. Data represent mean ± SEM.
